# mSphere of Influence: Role of IRGM1 in Disease Control

**DOI:** 10.1128/mSphere.00252-21

**Published:** 2021-04-14

**Authors:** Sumanta Kumar Naik

**Affiliations:** a Department of Molecular Microbiology, Washington University in St. Louis School of Medicine, St. Louis, Missouri, USA

**Keywords:** IRGM1, *Mycobacterium tuberculosis*, mitophagy, type I interferon

## Abstract

Sumanta K. Naik works in the tuberculosis field, with a specific interest in the host immune response to Mycobacterium tuberculosis infection. In this mSphere of Influence article, he reflects on how the paper “IRGM1 links mitochondrial quality control to autoimmunity” by Prashant Rai et al. (Nat Immunol, 22:312–321, 2021, https://doi.org/10.1038/s41590-020-00859-0) impacted his research by revealing new roles for Irgm1 in immune responses.

## COMMENTARY

Before starting my postdoctoral training period, my impression was that the interferon-regulated GTPase IRGM1 was required for autophagy and deficiency in IRGM1 and that it leads to defects in autophagosome-lysosome fusion, which contributes to severity in the outcome of infectious and autoimmune diseases. One such widespread infectious disease shown to require IRGM1 for host immune control is tuberculosis (TB), which is caused by the pathogenic bacterium Mycobacterium tuberculosis. M. tuberculosis can withstand immune responses and antibiotic therapy, and as a result, regimens for treating active TB disease involve at least 6 months of anti-TB drugs, which can have adverse side effects on human health. Hence, harnessing the host immune response through host-directed therapies has become an attractive option for treating M. tuberculosis infections. My postdoctoral research focuses on dissecting the host immune response to gain insight into possible host-directed therapeutic strategies for the treatment of TB, which involves studying different host genes that play vital roles in the immune response to M. tuberculosis infection. Mice deficient in *Irgm1* have been found to be susceptible to M. tuberculosis infection and fail to control M. tuberculosis replication. Initial reports presented a model where IRGM1 contributes to M. tuberculosis clearance through autophagy, targeting of the bacteria to the lysosome for degradation, a process termed xenophagy (1, 2). However, subsequent publications reported that IRGM1 does not colocalize with mycobacteria containing phagosomal vacuoles (3) and that xenophagy does not contribute to the control of M. tuberculosis replication (4). These newer findings highlight that our understanding regarding the role for IRGM1 in TB disease control is still limited.

A recent publication by Rai et al. (5) has revealed that Irgm1 has a role in mitophagy, the autophagic clearance of damaged mitochondria, and that it controls type I interferon (IFN-I) responses and prevents autoimmunity. To study the cellular mechanism of IFN-I induction, Rai et al. treated *Irgm1^+/+^* and *Irgm1^−/−^* murine embryonic fibroblasts (MEFs) with IFN-γ and found that the treated *Irgm1^−/−^* MEFs have increased expression of interferon-stimulated genes (ISGs), which was abolished in *Irgm1^−/−^ Ifnar^−/−^* MEFs. These findings suggested that endogenous interferon-related stimuli, such as DNA, RNA, or mitochondrial DNA (mtDNA), can induce the IFN-I response in *Irgm1^−/−^* MEFs. The authors found increased mtDNA copy numbers in the cytosol of *Irgm1^−/−^* MEFs, which can be a result of defective mitophagy. *Irgm1^+/+^* and *Irgm1^−/−^* MEFs showed increased autophagy upon starvation, indicating that *Irgm1^−/−^* MEFs are autophagy competent under high-stress conditions. However, with IFN-γ stimulation only, multiple mitochondrial abnormalities (such as increased mitochondrial reactive oxygen species, increased depolarized mitochondria, and reduced colocalization of Rab5 and mitochondrial protein HSP60) were observed in *Irgm1^−/−^* MEFs, suggesting a defect in mitophagy in comparison to that in *Irgm1^+/+^* MEFs. These findings suggest that a defect in the endosomal transfer of mitochondria containing autophagosomes (mitoAPs) to the lysosome caused an increased accumulation of depolarized mitochondria. Accumulation of depolarized mitochondria allowed the mtDNA access to the cytosol, which induced IFN-I signaling via the double-stranded DNA receptor cGAS. Differences in lysosomal biogenesis can affect mitophagy and IFN-I induction, but there were no differences in lysosomal biogenesis in *Irgm1^+/+^* and *Irgm1^−/−^* MEFs. However, the LysoTracker signal was reduced in *Irgm1^−/−^* MEFs, indicating a failure in the fusion of mitoAPs with degradative lysosomes, thus impairing mitophagy and inducing IFN-I responses. The authors also studied whether the same mechanism was happening in macrophages using bone marrow-derived macrophages (BMDMs). Remarkably, in comparison to MEFs, *Irgm1^−/−^* BMDMs showed increased colocalization of mitoAPs with acidified lysosomes but abnormal accumulation of the autophagy protein LC3. *Irgm1^−/−^* BMDM displayed an increased IFN-I response that was cGAS independent but STING dependent, where deletion of *Tmem173*, which encodes STING, rescued the heightened IFN-I response. Silencing of mitophagy-associated kinase PINK1 and endosomal Toll-like receptor TLR7 resulted in the downregulation of ISGs in *Irgm1^−/−^* BMDM, suggesting the involvement of the PINK1-LC3 pathway in delivering the mtDNA to TLR7 to initiate the IFN-I response. Together, these data indicate cell-specific roles for Irgm1 repressing the induction of the IFN-I response.

Enhanced IFN-I signaling can worsen autoimmune diseases and bacterial infections, particularly in the case of TB. Mitochondrial DNA released into the cytosol has been reported to induce IFN-I responses during M. tuberculosis infection (6). Increased IFN-I responses contribute to M. tuberculosis pathogenesis by increasing interleukin 10 (IL-10) anti-inflammatory responses and decreasing IFN-γ responses (7). Therefore, this raises the possibility that Irgm1 may be required to control M. tuberculosis infection by regulating IFN-I responses. Granulomas are the primary setting of pulmonary TB and are comprised of different cell types, such as neutrophils, macrophages, lymphocytes, and fibroblasts. Rai et al. (5) demonstrated that *Irgm1* can induce immune responses differently in two different types of cells. Considering this, IRGM1 likely has diverse roles in the different immune cells within a TB granuloma, which will need to be investigated during different stages of TB disease progression. For the treatment of TB disease, preventing defective mitochondrial abnormalities by enhancing the activity conferred by IRGM1 may be a powerful approach to obtain host-directed therapies.

## References

[1] MacMicking JD, Taylor GA, McKinney JD. 2003. Immune control of tuberculosis by IFN-gamma-inducible LRG-47. Science 302:654–659. doi:10.1126/science.1088063. 14576437

[2] Singh SB, Davis AS, Taylor GA, Deretic V. 2006. Human IRGM induces autophagy to eliminate intracellular mycobacteria. Science 313:1438–1441. doi:10.1126/science.1129577. 16888103

[3] Springer HM, Schramm M, Taylor GA, Howard JC. 2013. Irgm1 (LRG-47), a regulator of cell-autonomous immunity, does not localize to mycobacterial or listerial phagosomes in IFN-γ-induced mouse cells. J Immunol 191:1765–1774. doi:10.4049/jimmunol.1300641. 23842753 PMC3753587

[4] Kimmey JM, Huynh JP, Weiss LA, Park S, Kambal A, Debnath J, Virgin HW, Stallings CL. 2015. Unique role for ATG5 in neutrophil-mediated immunopathology during M. tuberculosis infection. Nature 528:565–569. doi:10.1038/nature16451. 26649827 PMC4842313

[5] Rai P, Janardhan KS, Meacham J, Madenspacher JH, Lin WC, Karmaus PWF, Martinez J, Li QZ, Yan M, Zeng J, Grinstaff MW, Shirihai OS, Taylor GA, Fessler MB. 2021. IRGM1 links mitochondrial quality control to autoimmunity. Nat Immunol 22:312–321. doi:10.1038/s41590-020-00859-0. 33510463 PMC7906953

[6] Wiens KE, Ernst JD. 2016. The mechanism for type I interferon induction by Mycobacterium tuberculosis is bacterial strain-dependent. PLoS Pathog 12:e1005809. doi:10.1371/journal.ppat.1005809. 27500737 PMC4976988

[7] Ji DX, Yamashiro LH, Chen KJ, Mukaida N, Kramnik I, Darwin KH, Vance RE. 2019. Type I interferon-driven susceptibility to Mycobacterium tuberculosis is mediated by IL-1Ra. Nat Microbiol 4:2128–2135. doi:10.1038/s41564-019-0578-3. 31611644 PMC6879852

